# Gut Microbiome Diversity and Abundance Correlate with Gray Matter Volume (GMV) in Older Adults with Depression

**DOI:** 10.3390/ijerph19042405

**Published:** 2022-02-19

**Authors:** Sungeun Melanie Lee, Michaela M. Milillo, Beatrix Krause-Sorio, Prabha Siddarth, Lisa Kilpatrick, Katherine L. Narr, Jonathan P. Jacobs, Helen Lavretsky

**Affiliations:** 1 Department of Psychiatry, Semel Institute for Neuroscience, University of California Los Angeles, 760 Westwood Plaza, Los Angeles, CA 90024, USA; sungeunl@gmail.com (S.M.L.); michaela.m.milillo@gmail.com (M.M.M.); bkrause@ucla.edu (B.K.-S.); psiddarth@mednet.ucla.edu (P.S.); lakilpatrick@mednet.ucla.edu (L.K.); 2 Brain Research Institute, 635 Charles E Young Drive South, Los Angeles, CA 90095, USA; narr@ucla.edu; 3 UCLA Microbiome Center, David Geffen School of Medicine at UCLA, 10833 Le Conte Ave., Los Angeles, CA 90095, USA; jjacobs@mednet.ucla.edu; 4 The Vatche and Tamar Manoukian Division of Digestive Diseases, Department of Medicine, David Geffen School of Medicine at UCLA, 10833 Le Conte Ave., Los Angeles, CA 90095, USA; 5 Division of Gastroenterology, Hepatology and Parenteral Nutrition, VA Greater Los Angeles Healthcare System and Department of Medicine and Human Genetics, 11301 Wilshire Blvd., Los Angeles, CA 90073, USA

**Keywords:** gray matter volume (GMV), gut–brain axis, geriatric depression (GD)

## Abstract

Growing evidence supports the concept that bidirectional brain–gut microbiome interactions play an important mechanistic role in aging, as well as in various neuropsychiatric conditions including depression. Gray matter volume (GMV) deficits in limbic regions are widely observed in geriatric depression (GD). We therefore aimed to explore correlations between gut microbial measures and GMV within these regions in GD. Sixteen older adults (>60 years) with GD (37.5% female; mean age, 70.6 (SD = 5.7) years) were included in the study and underwent high-resolution T1-weighted structural MRI scanning and stool sample collection. GMV was extracted from bilateral regions of interest (ROI: hippocampus, amygdala, nucleus accumbens) and a control region (pericalcarine). Fecal microbiota composition and diversity were assessed by 16S ribosomal RNA gene sequencing. There were significant positive associations between alpha diversity measures and GMV in both hippocampus and nucleus accumbens. Additionally, significant positive associations were present between hippocampal GMV and the abundance of genera Family_XIII_AD3011_group, unclassified Ruminococcaceae, and Oscillibacter, as well as between amygdala GMV and the genera Lachnospiraceae_NK4A136_group and Oscillibacter. Gut microbiome may reflect brain health in geriatric depression. Future studies with larger samples and the experimental manipulation of gut microbiome may clarify the relationship between microbiome measures and neuroplasticity.

## 1. Introduction

Major depression affects over 265 million people worldwide [1]. Major depressive disorder (MDD) is a disabling mental illness characterized by consistent mood changes accompanied by a variety of symptoms such as sadness, sleep disturbances, anhedonia, disturbances in appetite and sexual functioning, and suicidal ideation [2]. Geriatric depression (GD) is a serious public health issue associated with high morbidity, mortality and suicide [3,4]. Compared to depression in younger adults, geriatric depression has worse outcomes marked by a lower treatment response and remission rates, and significant cognitive and physical comorbidities [5,6]. More accurate multi-modal biomarker models to further our understanding of the interaction of different biological systems relating to GD are urgently needed for the development of novel treatment approaches.

There is a known relationship between depression, brain aging and neurodegenerative disorders. GD is a known risk factor for developing Alzheimer’s disease, a devastating neurodegenerative disease associated with the hippocampal atrophy and cognitive decline [7,8]. While limbic regions regulating emotions, which include the hippocampus and the amygdala, demonstrate volume reduction in depression across all ages, posterior cortical regions (e.g., the occipital cortex) appear to be spared [9]. Lower GMV in fronto-limbic regions, including the medial frontal gyri, prefrontal cortices, hippocampus, and amygdala have been found in both younger and older adults with depression compared to healthy controls [10,11,12,13,14,15]. Age-related brain atrophy with a reduction in GMV has also been indicated in older adults with cognitive impairment [16,17,18,19,20]. Positive responses to antidepressant treatment in depression have led to increases in GMV along with improvements in depression severity [21,22,23]. Furthermore, orbitofrontal volume can distinguish non-remitted from remitted patients with depression, suggesting a neurobiological prediction model of GMV for GD [24]. Additionally, genetic risk alleles such as APOE-4 are associated with GMV in geriatric depression, including cerebrovascular and neurodegenerative changes [25,26].

In addition to the existing neuroimaging data, an emerging and promising area of biological GD mechanisms is that of gut microbiota [27]. The gut microbiota, as the largest reservoir of microbes in the human body, have an essential role in the central nervous system (CNS) functioning through the bidirectional gut–brain axis [28,29,30]. In particular, gut dysbiosis has been linked to several mental health disorders including depression, anxiety, schizophrenia, and insomnia, as well as neurodegenerative disorders such as Parkinson’s disease and Alzheimer’s disease [31,32,33,34,35,36,37,38,39,40,41]. The literature on gut dysbiosis associated with GD compared to age-matched healthy controls is limited, but the meta-analysis of several case-control studies of younger adults with MDD shows a decreased abundance of genera Faecalibacterium, Dialister, Bifidobacterium, Escherichia/Shigella and Ruminococcus [42,43,44,45]. Interestingly, in a cross-sectional study of a large population cohort, Faecalibacterium was associated with higher quality of life indicators [46]. Several different mechanisms of how microbiota modulates the gut–brain axis have been described. This includes the regulation of the hypothalamus–pituitary–adrenal (HPA) axis, signaling through the vagal nerve, as well as producing molecular candidates such as neuropeptides, endocrine hormones, neurotransmitters and immunomodulators [28,47].

Gut microbiota composition and diversity change across the lifespan and are likely responsible for higher susceptibility to immune weaknesses and diseases at older ages [48]. We previously demonstrated that antidepressant treatment response in GD was predicted by an enrichment in Faecalibacterium, Agathobacter, and Roseburia: individual taxa which significantly distinguished remitters from non-remitters [27]. In addition, we previously found treatment-related changes in differential abundance in remitters. Specifically, Flavonifractor was significantly increased, while Roseburia was significantly decreased after successful antidepressant treatment. On the contrary, there were no significant changes in the non-remitter group. These results suggest a role for gut microbiota in predicting and modulating an antidepressant treatment response which seems to parallel the known correlation between GMV and depression, though the relationship between the gut microbiota and GMV has not been directly investigated in GD. In this exploratory cross-sectional study, we investigated correlations between GMV in subcortical limbic regions of interest that are widely implicated in depression pathophysiology with the abundance and diversity of the gut microbiota in elderly subjects with major depression.

## 2. Materials and Methods

### 2.1. Participants 

Participants were recruited from geriatric ambulatory care settings (UCLA Geriatric Medicine-Psychiatry clinics) and from local advertisements to participate in the parent clinical trial of levomilnacipran for geriatric depression (NCT 02466958) that was previously reported elsewhere [27,49]. Of 29 randomized patients, 16 older adults (37.5% female; mean age, 70.6 (SD = 5.7) years; Table 1) completed both MRI and stool sample collection and were included in this report. Inclusion criteria included age of 60 years or older with a diagnosis of MDD and a Mini-Mental State Examination (MMSE) score of >24 (absence of mild cognitive impairment or dementia). MDD criteria were assessed via the Structured Clinical Interview DSM-5 (SCID-5) [50]. Depressive symptom severity was assessed using the Hamilton Depression Rating Scale (HAMD-24) [51,52] and the Montgomery–Åsberg Depression Rating Scale (MADRS) [53]. Exclusion criteria and recruitment have previously been described in more detail [49]. This study was approved by the UCLA Institutional Review Board. All participants signed an informed consent form prior to the initiation of assessments.

### 2.2. MRI

At the Ahmanson-Lovelace Brain Mapping Center at UCLA, T1-weighted images (3D multi-echo magnetization prepared rapid gradient-echo sequences, MEMPRAGE) were acquired using a Siemens 3T Prisma system (Siemens, Erlangen, Germany) and a 32-channel head coil. The acquisition parameters were as follows: echo times = 1.74, 3.6, 5.46, and 7.32 ms; repetition time = 2530 ms; inversion time = 1260 ms; flip angle = 7 degrees; voxel size = 1 mm^3^; double GRAPPA and matrix size = 256 × 192; total acquisition time = 5:18 min. We used Freesurfer 6.0 (http://surfer.nmr.mgh.harvard.edu/ accessed on 6 January 2022) for cortical surface reconstruction. Preprocessing included the correction of magnetic field inhomogeneities, removal of nonbrain tissues from the images, cortical parcellation, and segmentation of subcortical gray matter. Cortical parcellation was based on the Desikan–Killiany atlas [54] and GMV was extracted from the following regions of interest: bilateral amygdala, hippocampus, nucleus accumbens, and the pericalcarine cortex, which served as a control region. Bilateral subcortical volumes were generated by the sums of left and right regional subcortical volumes.

### 2.3. Microbial Analysis

#### 2.3.1. Intestinal Microbial Composition: Stool Collection, Processing, and Analysis of 16S rRNA Gene Sequencing Data

Stool specimens were collected at home, stored in a home freezer, and returned to UCLA on cold packs for storage in an RNA stabilizing reagent (RNALater) at −80 °C. Genomic DNA was extracted from fecal samples using the Powersoil kit and bead beating as per the manufacturer’s instructions (QIAGEN, Germantown, MD, USA) [55]. 16S ribosomal RNA (rRNA) gene sequence libraries were generated with the fecal genomic DNA as a template for polymerase chain reaction (PCR) amplification of the V4 hypervariable region with barcoded primers (F515/R806) [56]. The Illumina MiSeq sequencing platform (San Diego, CA, USA) was used to perform paired-end sequencing (2 × 250 bp). DADA2 algorithm was used to perform quality filtering, merge paired-end reads, remove chimeras, and cluster sequences into exact amplicon sequence variants (ASVs) [57]. Taxonomic assignments were performed using the Silva database version 132 as a reference [58]. Sequence depth ranged from 36,341 to 113,025 sequences per sample with a total of 2245 assigned ASVs. Output sequences were classified at the domain, phylum, family, genus, and species levels where possible, depending on the depth of reliable classifier assignments [41].

#### 2.3.2. Within-Sample Diversity Analysis

Alpha-diversity metrics (i.e., bacterial diversity within a sample) were assessed in QIIME using Faith’s phylogenetic diversity (fraction of a phylogenetic tree represented in each sample), Chao1 (species richness), and the Shannon index (species evenness and richness) with the data were rarefied to 36,341 sequences. Associations between alpha-diversity metrics and regional GMV were tested using multivariable linear regression models with standardized regression coefficients (β), which facilitated comparison across models. The selection of covariates for the regression analyses relied on the univariate testing of demographic and clinical variables in association with regional GMV (Table S1) and alpha diversity metrics (Table S2). Pearson’s correlations or *t*-tests were used and significance was determined at an alpha level of 5% (*p* < 0.05). Due to the small sample size, Cohen’s d was utilized as an additional covariate selection criterion for binary variables (e.g., sex) (|d| > 0.8).

#### 2.3.3. Between-Sample Diversity Analysis

β-diversity assessment was performed by compositional distance metric based on robust Aitchison distances implemented with the DEICODE plugin in QIIME2 and visualized with principal coordinates analysis [59]. ASV count data were filtered to remove ASVs present in less than 12.5% of the samples. The statistical significance of differences in β-diversity in association with regional GMV was assessed using Adonis, a permutational multivariate analysis of variance using distance matrices, implemented in the Vegan package in R [60].

#### 2.3.4. Differential Taxonomic Abundance Analysis

Differential abundance of microbial genera in association with regional GMV was determined using multivariate negative binomial mixed models using DESeq2 in R [61]. The unrarefied genus counts were normalized by the size factor (median value of all ratios for a given sample). After filtering genera present in less than fifty percent of the sample size, a total of 81 genera were tested. The Benjamini–Hochberg false discovery rate (FDR) correction was used to adjust for multiple hypothesis testing and a significant association was defined at the FDR q-value < 0.05 [62]. Each individual genus identified from the DESeq2 analysis (q < 0.05) was then tested for associations with regional GMV using linear regression analyses and adjusted for age and sex (*p* < 0.05). The unrarefied genus counts were regularized log transformed (rlog), which normalizes the count with respect to the library size and minimizes differences between samples with low counts of the taxon.

## 3. Results

### 3.1. Participant Characteristics

A total of 41 older adults with GD were recruited, and sixteen (37.5% women, mean age = 70.6 years, SD = 5.7) completed both brain imaging and stool sample collection for microbiota analysis (Table 1). All but one participant was Caucasian (data not shown). The mean HAMD score was 18.6 (SD = 2.4) and the mean MADRS score was 14.6 (SD = 3.6).

### 3.2. Gut Microbiota Diversity Association with GMV

Univariate testing revealed significant negative correlations between age and hippocampal (R = −0.57, *p* = 0.02), amygdala (R = −0.69, *p* = 0.003) and nucleus accumbens volumes (R = −0.66, *p* = 0.006; Table S1). Age correlated negatively with alpha diversity measures, Chao1 (R = −0.54, *p* = 0.03) and Faith’s PD (R = −0.5, *p* = 0.05; Table S2). Additionally and as expected, there were large, albeit non-significant, differences between men and women in amygdala (D = 0.92, *p* = 0.09) and nucleus accumbens volumes (D = 0.97, *p* = 0.08) (Table S1). Consequently, we selected age and sex as covariates in the subsequent analyses of associations between microbiota composition and GMV.

After controlling for age and sex, we found significant positive associations between hippocampus volume and alpha diversity (community diversity within subjects) including Chao1 index (β = 0.571, *p* = 0.032) and Faith’s PD (β = 0.617, *p* = 0.028). We also found significant positive associations between nucleus accumbens volume and all three measures of alpha diversity, including Chao1 index (β = 0.791, *p* = 0.006), Faith’s PD (β = 0.752, *p* = 0.018) and Shannon index (β = 0.809, *p* = 0.020). However, what appeared as a positive association between amygdala volume and alpha diversity measures upon visual inspection, was not statistically significant after correcting for age and sex (Table 2 and Figure S1). There was no association between alpha diversity and GMV in the control region (Table 2 and Figure S1).

**Figure 1:**
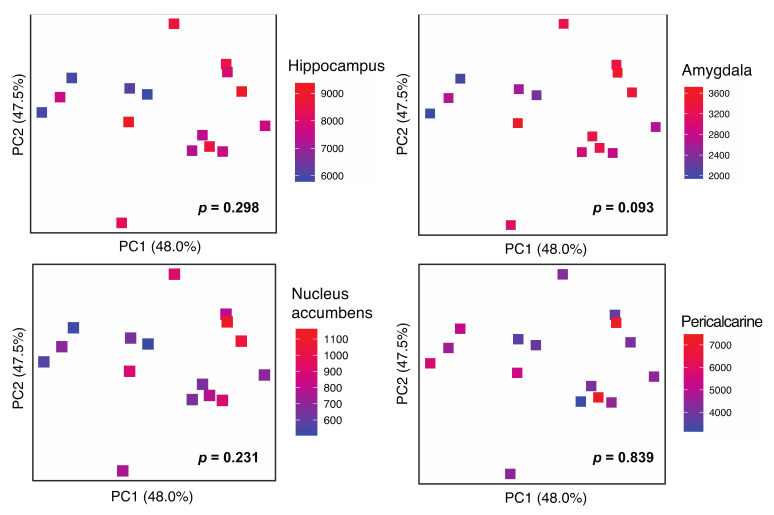
Gray matter volume (GMV) association with fecal microbial β-diversity. GMV regions include hippocampus, amygdala, nucleus accumbens and pericalcarine (control). Microbial β-diversity measured by robust Aitchison distance is visualized by principal coordinates analysis (PCoA) plots. Each symbol represents a sample with voxel-based GMV represented by the color gradient. *p*-values for microbial β-diversity association with GMV are calculated by Adonis adjusting for age and sex.

Beta diversity (community diversity between subjects) testing by robust Aitchison PCA revealed a graphical change in the association with amygdala volume, but did not reach statistical significance after adjusting for age and sex (Adonis *p* = 0.093; Figure 1). GMV in the hippocampus, nucleus accumbens and pericalcarine cortex (control) did not demonstrate associations with the microbiota beta diversity.

### 3.3. Individual Taxa Association with Gray Matter Volumes

**Figure 2:**
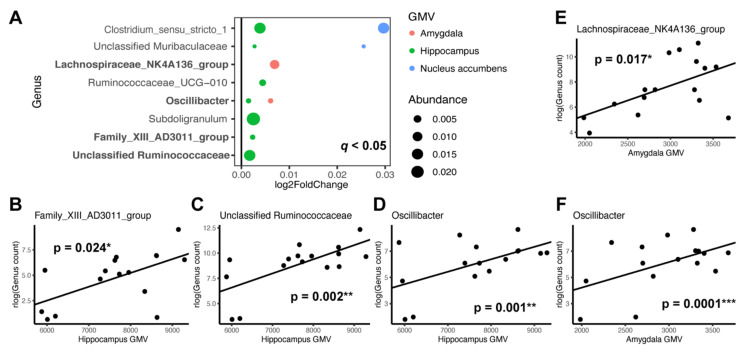
Specific microbial genera associated with GMV regions. GMV regions tested include hippocampus, amygdala, nucleus accumbens and pericalcarine (control). (**A**) DESeq2 was used to identify microbial genera associated with GMV with false discovery rate of q < 0.05 after adjusting for age and sex. Log2 fold change represents effect size and direction of these associations. Dot size is proportional to the relative abundance of the genus and color corresponds to the specific brain region. Bolded *y*-axis text: genera that were confirmed by individual testing of the taxon to be significantly associated with a specific GMV. (**B**–**F**) Scatter plots displaying GMV of a specific brain region and regularized log transformed (rlog) normalized count of individual genus with significant associations after adjusting for age and sex (*p* < 0.05). Regression lines are drawn without controlling for age and sex. * *p* < 0.05. ** *p* < 0.01. *** *p* < 0.001.

We performed differential abundance testing to identify the specific genera of gut microbiota that were associated with regional GMV in GD. After generating genus-level taxonomic summaries in QIIME, we conducted a multivariate DESeq2 analysis and controlled for age and sex. This revealed that seven individual genera were positively associated with hippocampal GMV, two with amygdala volume and two with nucleus accumbens volume at a false discovery rate of q < 0.05 (Figure 2A). Subsequently, we tested these genera individually with the corresponding regional GMV using a linear regression model with age and sex as covariates (Table S3). Out of the seven genera tested, genus *Family_XIII_AD3011_group* (β = 0.7, *p* = 0.024), an unclassified *Ruminococcaceae* (β = 0.856, *p* = 0.002), and genus *Oscillibacter* (β = 0.805, *p* = 0.001) demonstrated significant positive associations with hippocampal volume (Figure 2B–D). Genera *Lachnospiraceae_NK4A136_group* (β = 0.897, *p* = 0.017) and *Oscillibacter* (β = 1.055, *p* = 0.00008) were positively associated with amygdala volume (Figure 2E,F). However, the two genera initially identified by DESeq2 to be associated with nucleus accumbens volume did not show significant associations when tested individually.

## 4. Discussion

### 4.1. Gut Microbiome Diversity and Brain Structures

We report significant positive associations between gut microbiome alpha diversity and hippocampal and nucleus accumbens volumes after controlling for age and sex. As for the specific alpha diversity measures, hippocampal volume associated significantly with Chao1 index (species richness) and Faith’s PD (phylogenetic diversity), while nucleus accumbens volume associated positively with Chao1 index, Faith’s PD and Shannon index (species evenness and richness). Amygdala volume and the pericalcarine region showed no associations with the alpha diversity metrics. Moreover, no significant relationship was found between beta diversity and any of the selected ROIs.

Age and sex are important variables in brain volumetric and gut microbiome research [63,64,65]. As previously reported [9,66], older age was associated with greater decline in the hippocampus, amygdala, and the nucleus accumbens (Table S1). We additionally found a significant negative correlation between age and Chao1 index, further suggesting that age is a confounding factor that needs to be adjusted for in examining the relationship between GMV and gut microbial diversity (Table S2). Although not statistically significant, we found smaller volumes of amygdala and nucleus accumbens brain regions in females (Table S1). We did not find significant relationships between clinical variables and GMV in our selected ROIs, noting our sample size might have precluded the detection of smaller effects.

In this pilot study, we focused on hippocampus, amygdala and nucleus accumbens as subcortical brain regions of interest, as they have been previously implicated in aging and depression. Healthy aging involves cortical thinning across the brain, with relative sparing of the lateral temporal cortex and the entorhinal cortex, while the hippocampus declines at increasingly higher rates with aging [66,67]. There appears to be a complex relationship between memory and hippocampal volume that is age- and test- dependent [68]. In depressed samples, hippocampus is consistently found to be reduced compared to non-depressed controls [69]. Moreover, such hippocampal decline can be detected even in first-episode depressed patients, including children as early as pre-school age [9,70], and linked to stress factors [71]. The amygdala and nucleus accumbens are additional limbic structures with a prominent role in depression [9]. Based on rodent research, it has been suggested that the nucleus accumbens, as a dopaminergic recipient within the mesolimbic loop, has a prominent role in depression and potentially its etiology [72].

Despite the paucity of literature on brain–gut communication in human neuropsychiatric disorders, there is one case-control study of 38 schizophrenia patients, directly investigating the association between gut microbial diveresity and GMV which found positive correlations between gut microbial alpha diversity and GMV of bilateral insula, right postcentral gyrus and left inferior operculum frontal cortex [73]. Of note, all patients showed decreased volumes in all regions compared to the controls. Our exploratory findings add to the increasing body of evidence demonstrating associations between gut microbiome and brain structure in neuropsychiatric disorders, wherein greater brain volumes and increased gut diversity measures appear to correlate with health. The positive association between the brain volumes of interest and gut microbial diversity is not only exciting from a mechanistic perspective in further elucidating the complex brain–gut–microbiota crosstalk, but it has a practical interventional implication, as it is possible to manipulate the gut microbiome through diet and probiotic supplementation as a conduit to influence the brain structure and function.

### 4.2. Microbial Taxa and Brain Structures

All of the reported positive associations between several individual taxa and hippocampal and amygdala volume in this study were members of Clostridiales at the order level, Clostridia at the class level, and Firmicutes at the phylum level. Of these, relative abundance of the genus Oscillibacter demonstrated highly significant and strongly positive associations with both hippocampal and amygdala volumes. At least two studies have shown significantly enriched Oscillibacter in MDD patients compared to healthy controls [43,74]. As cross-sectional studies are unable to establish causal links between individual taxa and the disorder under investigation, it is unclear whether increased Oscillibacter contributed to causing depression, or whether depression of the host brought on the ‘blooming’ of Oscillibacter in the gut. Although we can only postulate, it is possible that an increased abundance of Oscillibacter may have conferred protective modulatory function to the brain that resulted in increased amygdala and hippocampal volumes in our cohort of GD patients. For example, the main metabolic end product of Oscillibacter is n-Valeric acid [75], which has a similar chemical structure to the neurotransmitter GABA. This is a possible pathway by which *Oscillibacter* can mediate beneficial effects on the brain as a psychobiotic agent. However, this idea remains to be investigated through mechanistic studies wherein manipulation of the gut microbiome can be causally linked to clinical as well as brain volumetric outcomes.

### 4.3. Limitations

There are several limitations to our pilot study, with the primary one being the small sample size. This was due to the fact that many older adults were ineligible for MRI scanning based on implants that were either unsafe to scan (e.g., pacemakers, hip or knee replacements), or where exact implant documentation was missing. Due to the small sample size, we were limited in the number of brain regions investigated given the risk of multiple testing and false positive results. We were also unable to ascertain any relationship between gut microbial measures and clinical variables such as depression severity, cognitive function, ethnicity, and life styles due to the small and homogenious study sample. Therefore, a larger sample size will be needed to overcome some of these limitations and also be able to test other potential brain regions of interest that are likely implicated in GD. Additionally, a longitudinal study will allow for an investigation of the gut–brain axis in terms of the relationship with depression risk, disease progression, and prognosis. However, our findings provide promising preliminary data demonstrating a potential relationship between the gut microbiota, at the community as well as individual taxonomic levels, and the volumetric brain correlates within the important regions of interest for GD, opening new avenues for future investigation into brain aging, gut health, and neural mechanisms of GD. This avenue may support the discovery of new targets for microbiome-focused interventions for the treatment and prevention of depression and cognitive decline in older adults.

## 5. Conclusions

This pilot study explored the associations between the volumetric measures of the gray matter volume in the limbic subcortical brain regions and fecal microbial diversity and composition in older adults with depression. We found significant positive associations between several alpha diversity measures and GMVs of hippocampus and nucleus accumbens. At the individual taxa level, we identified four genera belonging to the order *Clostridiales* that were significantly positively associated with the GMV of either hippocampus or amygdala. Of these, genus *Oscillibacter* demonstrated highly significant and strongly positive associations with both hippocampal and amygdala volumes, suggesting a potentially beneficial or protective role of *Oscillibacter* in GD. This study adds to the growing literature highlighting the bidirectional communication along the gut microbiome–brain axis. To our knowledge, this is the first study directly investigating and providing a proof of concept for the relationship between the brain structural measures and the gut microbiota composition in otherwise healthy older adults with depression.

## Figures and Tables

**Table 1:** Demographic and clinical characteristics of the participants with GD.

Variables ^1^	Cohort (*N* = 16)
Age (years), mean +/− SD	70.6 +/− 5.7
Female, n (%)	6 (37.5)
Education (years), mean +/− SD	16.0 +/− 1.5
Age of onset (years), mean +/− SD	47.2 +/− 25.0
BMI (kg/m^2^), mean +/− SD	26.6 +/− 3.6
MMSE, mean +/− SD	28.9 +/− 3.6
MADRS, mean +/− SD	14.6 +/− 3.6
HAMD, mean +/− SD	18.6 +/− 2.4

^1^ BMI = Body mass index; MMSE = Mini-Mental State Examination; MADRS = Montgomery-Åsberg Depression Rating Scale; HAMD = Hamilton Depression Rating Scale (24 item).

**Table 2:** Fecal microbial alpha diversity association with regional GMV by multivariable linear regression with age and sex as covariates. Alpha diversity measures included Chao1, Faith’s PD, and Shannon index. GMV regions included hippocampus, amygdala, nucleus accumbens and pericalcarine. β = standardized regression coefficient. *p*-value less than 0.05 are bolded. * *p* < 0.05. ** *p* < 0.01.

GMV Region	Chao1		Faith’s PD		Shannon	
	β	*p*	β	*p*	β	*p*
Hippocampus	**0.571**	0.032 *	**0.617**	0.028 *	0.528	0.097
Amygdala	0.516	0.116	0.495	0.159	0.401	0.303
Nucleus accumbens	**0.791**	0.006 **	**0.752**	0.018 *	**0.809**	0.020 *
Pericalcarine (control)	−0.148	0.554	−0.256	0.328	0.173	0.549

## Data Availability

For data availability, please contact the authors.

## Supplementary Materials

The following supporting information can be downloaded at: www.mdpi.com/article/10.3390/ijerph19042405/s1. Figure S1: Scatter plots of GMV association with fecal microbial alpha diversity as measured by Chao1, Faith’s PD, and Shannon index. GMV regions include hippocampus, amygdala, nucleus accumbens and pericalcarine (control). Table S1: Univariable testing of individual variable association with regional GMV. Table S2: Univariable testing of individual variable association with alpha diversity measures. Table S3: Individual microbial genera association with regional GMV by multivariable linear regression with age and sex as covariates.
