# Supplementary material for: Gut Microbiome Diversity and Abundance Correlate with Gray Matter Volume (GMV) in Older Adults with Depression
Source: Int J Environ Res Public Health. 2022 Feb 19;19(4):2405. doi: 10.3390/ijerph19042405 (PMC8872347; doi:10.3390/ijerph19042405)

Figure S1. Scatter plots of GMV association with fecal microbial alpha diversity as measured by Chao1, Faith's PD, and Shannon index. GMV regions include hippocampus, amygdala, nucleus accumbens and pericalcarine (control).

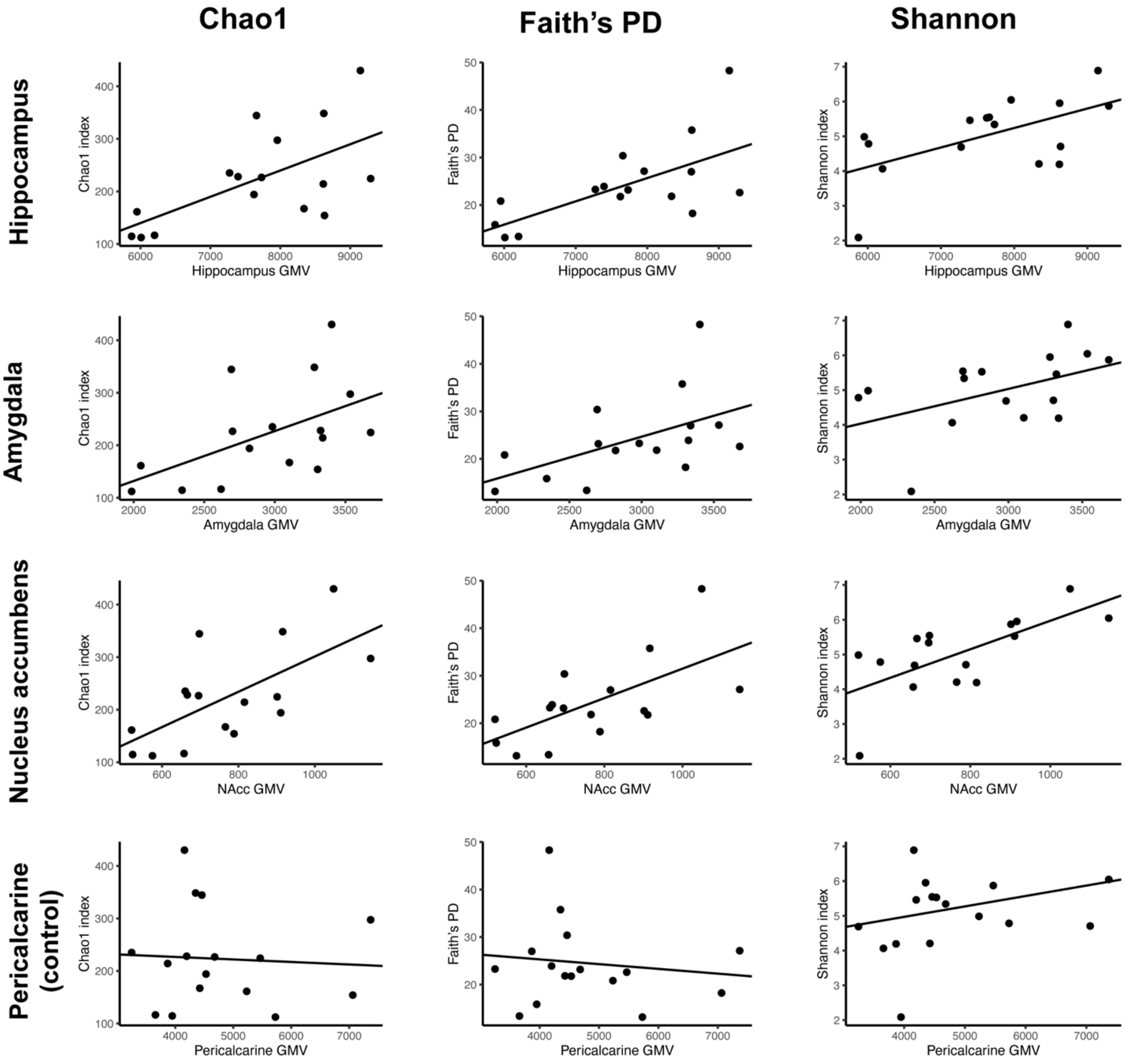

Supplement: Supplementary file 1 [file ijerph-19-02405-s001.zip › Figure S1.pdf]
