# Supplementary material for: Gut Microbiome Diversity and Abundance Correlate with Gray Matter Volume (GMV) in Older Adults with Depression
Source: Int J Environ Res Public Health. 2022 Feb 19;19(4):2405. doi: 10.3390/ijerph19042405 (PMC8872347; doi:10.3390/ijerph19042405)
Supplement: Supplementary file 1 [file ijerph-19-02405-s001.zip › Table S1.pdf]

Table S1. Univariable testing of individual variable association with regional GMV.

Variable	Hippocampus	Amygdala	Nucleus Accumbens	Pericalcarine
Age	**R = -.57**<br>**p = .02**	**R = -.69**<br>**p = .003**	**R = -.66**<br>**p = .006**	R = -.26<br>p = .33
Sex	D = -.67<br>p = .22	**D = -.92**<br>**p = .09**	**D = -.97**<br>**p = .08**	D = -.70<br>p = .17
Education	R = -.05<br>p = .85	R = .16<br>p = .55	R = .005<br>p = .99	R = .22<br>p = .41
Age of Onset	R = -.42<br>p = .1	R = -.4<br>p = .12	R = -.43<br>p = .1	R = -.03<br>p = .91
BMI	R = -.02<br>p = .94	R = .22<br>p = .42	R = .11<br>p = .68	R = -.32<br>p = .23
MMSE	R = .07<br>p = .8	R = .04<br>p = .89	R = -.06<br>p = .83	R = -.36<br>p = .16
MADRS	R = -.28<br>p = .29	R = -.23<br>p = .39	R = -.27<br>p = .31	R = -.13<br>p = .64
HAMD	R = .08<br>p = .76	R = .14<br>p = .61	R = .24<br>p = .36	R = -.02<br>p = .95

R=Pearson's correlation coefficient; D=Cohen's d; BMI = Body Mass Index; MMSE = Mini-Mental State Examination; MADRS = Montgomery-Åsberg Depression Rating Scale; HAMD = Hamilton Depression Rating Scale (24 item). P-values calculated by t-test or Pearson's correlation. Bolded: P-value less than 0.05 or absolute D-value greater than 0.8.
