# Supplementary material for: Gut Microbiome Diversity and Abundance Correlate with Gray Matter Volume (GMV) in Older Adults with Depression
Source: Int J Environ Res Public Health. 2022 Feb 19;19(4):2405. doi: 10.3390/ijerph19042405 (PMC8872347; doi:10.3390/ijerph19042405)
Supplement: Supplementary file 1 [file ijerph-19-02405-s001.zip › Table S2.pdf]

Table S2. Univariable testing of individual variable association with alpha diversity measures.

Variable	Chao1	Faith's PD	Shannon
Age	**R = -.54**<br>**p = .03**	R = -.5<br>p = .05	R = -.39<br>p = .14
Sex	D = .26<br>p = .62	D = .13<br>p = .79	D = -.35<br>p = .54
Education	R = -.27<br>p = .32	R = -.38<br>p = .15	R = -.17<br>p = .54
Age of Onset	R = -.51<br>p = .05	R = -.49<br>p = .06	R = -.09<br>p = .75
BMI	R = -.21<br>p = .43	R = -.06<br>p = .82	R = -.38<br>p = .15
MMSE	R = .17<br>p = .52	R = .21<br>p = .43	R = -.25<br>p = .36
MADRS	R = -.01<br>p = .96	R = -.03<br>p = .93	R = -.21<br>p = .44
HAMD	R = .11<br>p = .69	R = .15<br>p = .58	R = -.19<br>p = .49

R=Pearson's correlation coefficient; D=Cohen's d; BMI = Body Mass Index; MMSE = Mini-Mental State Examination; MADRS = Montgomery-Åsberg Depression Rating Scale; HAMD = Hamilton Depression Rating Scale (24 item). P-values calculated by t-test or Pearson's correlation. Bolded: P-value less than 0.05 or absolute D-value greater than 0.8.
