# Supplementary material for: Gut Microbiome Diversity and Abundance Correlate with Gray Matter Volume (GMV) in Older Adults with Depression
Source: Int J Environ Res Public Health. 2022 Feb 19;19(4):2405. doi: 10.3390/ijerph19042405 (PMC8872347; doi:10.3390/ijerph19042405)
Supplement: Supplementary file 1 [file ijerph-19-02405-s001.zip › Table S3.pdf]

Table S3. Individual microbial genera association with regional GMV by multivariable linear regression with age and sex as covariates.

Brain Region	Genus	β	p-value
Hippocampus	p__Bacteroidetes; c__Bacteroidia; o__Bacteroidales; f__Muribaculaceae; g__	.421	.189
Hippocampus	p__Firmicutes; c__Clostridia; o__Clostridiales; f__Clostridiaceae_1; g__Clostridium_sensu_stricto_1	.443	.097
**Hippocampus**	**p__Firmicutes; c__Clostridia; o__Clostridiales; f__Family_XIII; g__Family_XIII_AD3011_group**	**.700**	**.024***
**Hippocampus**	**p__Firmicutes; c__Clostridia; o__Clostridiales; f__Ruminococcaceae; g__**	**.856**	**.002****
**Hippocampus**	**p__Firmicutes; c__Clostridia; o__Clostridiales; f__Ruminococcaceae; g__Oscillibacter**	**.805**	**.001****
Hippocampus	p__Firmicutes; c__Clostridia; o__Clostridiales; f__Ruminococcaceae; g__Ruminococcaceae_UCG-010	.483	.154
Hippocampus	p__Firmicutes; c__Clostridia; o__Clostridiales; f__Ruminococcaceae; g__Subdoligranulum	.559	.090
**Amygdala**	**p__Firmicutes; c__Clostridia; o__Clostridiales; f__Lachnospiraceae; g__Lachnospiraceae_NK4A136_group**	**.897**	**.017***
**Amygdala**	**p__Firmicutes; c__Clostridia; o__Clostridiales; f__Ruminococcaceae; g__Oscillibacter**	**1.055**	**.00008*****
NAcc	p__Bacteroidetes; c__Bacteroidia; o__Bacteroidales; f__Muribaculaceae; g__	.361	.336
NAcc	p__Firmicutes; c__Clostridia; o__Clostridiales; f__Clostridiaceae_1; g__Clostridium_sensu_stricto_1	.461	.138

Normalized count of individual genera identified from DESeq2 analysis (depicted in Figure 2A) were regularized log transformed (rlog) for the linear regression. GMV regions include hippocampus, amygdala, and nucleus accumbens. β = standardized regression coefficient. P-value less than 0.05 uncorrected are bolded.
